# Soil Bacterial Community Structure Responses to Precipitation Reduction and Forest Management in Forest Ecosystems across Germany

**DOI:** 10.1371/journal.pone.0122539

**Published:** 2015-04-14

**Authors:** Katja Felsmann, Mathias Baudis, Katharina Gimbel, Zachary E. Kayler, Ruth Ellerbrock, Helge Bruehlheide, Johannes Bruckhoff, Erik Welk, Heike Puhlmann, Markus Weiler, Arthur Gessler, Andreas Ulrich

**Affiliations:** 1 Institute for Landscape Biogeochemistry, Leibniz Centre for Agricultural Landscape Research (ZALF), Müncheberg, Germany; 2 Institute of Biology/Geobotany and Botanical Garden, Martin Luther University Halle-Wittenberg, Halle, Germany; 3 Faculty of Environment and Natural Resources, University of Freiburg, Freiburg, Germany; 4 Department of Soils and Environment, Forest Research Institute Baden-Württemberg, Freiburg, Germany; 5 Berlin-Brandenburg Institute of Advanced Biodiversity Research (BBIB), Berlin, Germany; 6 Swiss Federal Institute for Forest, Snow and Landscape Research (WSL), Birmensdorf, Switzerland; Catalan Institute for Water Research (ICRA), SPAIN

## Abstract

Soil microbial communities play an important role in forest ecosystem functioning, but how climate change will affect the community composition and consequently bacterial functions is poorly understood. We assessed the effects of reduced precipitation with the aim of simulating realistic future drought conditions for one growing season on the bacterial community and its relation to soil properties and forest management. We manipulated precipitation in beech and conifer forest plots managed at different levels of intensity in three different regions across Germany. The precipitation reduction decreased soil water content across the growing season by between 2 to 8% depending on plot and region. T-RFLP analysis and pyrosequencing of the 16S rRNA gene were used to study the total soil bacterial community and its active members after six months of precipitation reduction. The effect of reduced precipitation on the total bacterial community structure was negligible while significant effects could be observed for the active bacteria. However, the effect was secondary to the stronger influence of specific soil characteristics across the three regions and management selection of overstorey tree species and their respective understorey vegetation. The impact of reduced precipitation differed between the studied plots; however, we could not determine the particular parameters being able to modify the response of the active bacterial community among plots. We conclude that the moderate drought induced by the precipitation manipulation treatment started to affect the active but not the total bacterial community, which points to an adequate resistance of the soil microbial system over one growing season.

## Introduction

Temperature as well as the variability of precipitation are expected to increase with climate change across Central Europe [1]. Current climate projections, based on the A1FI scenario, predict a 15% to 50% reduction of summer precipitation in Central Europe [1], with potentially severe consequences for tree vitality and growth as well as for biogeochemical cycles in forest ecosystems [2–4].

While special attention has been given to tree responses to drought, including processes related to xylem and leaf hydraulics as well as carbon uptake, storage and transport [5–8], belowground processes have rather been out of focus [9]. Soil microbes are key players in nutrient mineralization, decomposition of organic material, and modification of the soil structure [10, 11], and are therefore pivotal to our understanding of how forest eco-physiological and biogeochemical trajectories might shift with ongoing precipitation reduction. The reduction of precipitation and decrease in soil water availability will be crucial for soil microbes and can even have a stronger impact than other consequences of global climate change such as increases in temperature and CO_2_ concentration [12].

In general, the reduction in forest soil moisture will force soil microbes to either avoid or tolerate drought while facing the additional challenge of finding nutrient and energy sources that become spatially less available [13]. A reduction in soil water availability and an increase in the intensity and frequency of drought periods can lead to reduced decomposition and microbial growth as well as to changes in the microbial community structure [14–16]. However, there is also evidence of microbial communities being resistant [17] to frequent soil drying as total microbial biomass, physiological properties or community composition were not affected after such treatments [18, 19], or the drought response may only occur in specific microbial groups [20].

Besides water availability, soil characteristics have direct and immediate effects on soil microbes and their community structure. The main drivers were identified as soil type, organic matter, pH and C/N ratio [17, 21, 22]. However, the community is also influenced by more general effects as the land use intensity [23]. In grassland ecosystems it was found that lower land use intensity results in higher bacterial diversity [24], but these finding might not be directly extrapolated to forest ecosystems. However, management intensity can be a central driver of the abiotic and biotic conditions in a forest. Forest management determines overstorey tree composition by planting and selective felling [25, 26], which changes stand meso- and microclimatic conditions [27, 28], understorey diversity [29, 30], carbon and nutrient input into the soil [31, 32], and the water balance of the soil-vegetation-atmosphere continuum; e.g. [33, 34]. Management decisions such as frequency of forest harvesting could thus significantly affect the soil microbial community; however, single-site studies suggest that the responses of soil microbes can be highly variable, from strongly responsive to negligible in some regions [35–37]. Tree species selection in forest management practice also has an impact on the microbial community because trees and the associated understorey vegetation can influence the microbial community through changes in pH, litter chemistry, root density, and carbon exudates [38, 39].

Plant-soil interactions might modulate the effect of drought on the soil microbial community [17]. As a consequence the impacts of reduced precipitation may strongly vary among forest ecosystems, with plant diversity and the composition of the aboveground plant community [19, 40, 41] and thus with forest management. The allocation of carbon within the plant to belowground compartments is affected by drought and this might result in indirect effects of plants and plant species on microbial carbon metabolism and drought response strategies [42, 43]. Therefore, elucidating the combined effect of precipitation reduction and forest management on the microbial community in forest soils is the cornerstone of this investigation.

To date, most investigations have focused on extreme drought events often followed by subsequent rewetting [18, 44] and a conceptual understanding of the reaction of soil microbial communities under reduced soil water availability is beginning to emerge [13]. We follow Smith [45] in her definition of an extreme climatic event (ECE). An ECE is defined as an episode or occurrence in which a statistically rare or unusual climatic period alters ecosystem structure and⁄or function well outside the bounds of what is considered typical or normal variability. This synthetic definition of an extreme climatic event (ECE) includes ‘extremeness’ in both the driver and the response. We simulated a statistically rare climate period by reducing precipitation over six months. This reduction of the precipitation resulted, in only a moderate soil drought—i.e. a moderate reduction of the soil moisture content—in the forest soils. Currently, little information is available about responses of microbial communities to such moderate droughts over a growing season, which may occur more frequently in the future, and consequently this is the focus of our present study.

The aim of our investigation was to assess the response of the soil bacterial community towards precipitation reduction and a combination of reduced precipitation and varying forest management intensity also inducing changes in the over- and understorey vegetation. In particular we sought to answer the question: Does the bacterial community have the potential to withstand the induced drought period of one growing season? The experiment took place in the three Biodiversity Exploratories [46], a research platform that investigates functional biodiversity, providing gradients in soil properties and climatic conditions. We characterized the total and metabolically active bacterial community by T-RFLP (Terminal Restriction Fragment Length Polymorphism) analysis and 454 pyrosequencing of the 16S rRNA gene after six month of precipitation reduction. We assumed that the bacterial community structure varies across the three exploratories and across the different forest management intensities. Moreover, we hypothesized that a reduced precipitation will also result in changes of the bacterial community structure and that the specific characteristics of the different exploratories and forest management intensity will modify the resistance of bacterial communities to the precipitation reduction treatment.

## Materials and Methods

### Study sites and experimental design

The study has been carried out at the three regions of the Biodiversity Exploratories: Schorfheide-Chorin (S) in the north-east, Hainich Dün (H) in the middle and Schwäbische Alb (A) in the south-west of Germany. The Biodiversity Exploratories are a large scale biodiversity experimental platform funded by the German Research Foundation (DFG) with the impact of management on biodiversity as its focus; www.biodiversity-exploratories.de [46]. Field work permits were issued by the responsible state environmental offices of Baden-Württemberg, Thüringen, and Brandenburg (according to § 72 BbgNatSchG). At each exploratory, we selected three forest plots with different management intensity: (1) a plot unmanaged for at least 60 years (bu), dominated by *Fagus sylvatica* L., the understorey of which represents the potential natural vegetation, (2) a managed plot (bm), dominated by *F*. *sylvatica* and (3) an intensively managed plot (cm), dominated by conifers; *Picea abies* at Schwäbische Alb and Hainich or *Pinus sylvestris* L. at Schorfheide (Table 1). The soils at the Schorfheide plots range from sandy loam to pure sand Cambisols, whereas the soils at Hainich plots are loamy to clayey textured Luvisols and Stagnosols. The soils at the Schwäbische Alb plots are rich in clay and have a high stone content (Cambisols and Leptosols); BExIS database [47]. At each of the nine plots (1000 m^2^), we randomly established four pairs of subplots. The subplots, each with an area of 3 m x 3 m, were established within the tree interspaces with equal distances to the surrounding trees. Four of them were equipped with roofs (3 m x 3 m) in January 2012 and four served as unroofed control subplots (S1 Fig). The distance between control and precipitation manipulated subplots was 3 m. The roofs were constructed on a 2 m high wood frame fitted with durable rain gutters (permanent precipitation reduction of 11%), which allow light and airflow penetration. The captured precipitation water was drained to one side of the roof and channelled out of the plot. Transparent roof elements (acrylic tiles with an area per tile of 0.52 m^2^) were mounted randomly between the rain gutters to reduce precipitation. The position of the tiles was changed and the total coverage adjusted once per month. Partial covering of the roofs began on March 1st 2012. The target precipitation reduction at the precipitation manipulation subplots corresponded to the lower 2.5 percentile of the annual precipitation from the last 40 years (1951–2011) for each exploratory. For the calculations of the precipitation amount of a drought year we used the precipitation data of climate stations next to our experimental plots (Schorfheide: DWD (German Weather Service) station Angermünde (DWD-ID 00164), Hainich: Erfurt-Bindersleben (DWD-ID 00487), Schwäbische Alb: Münsingen/Apfelstetten (DWD-ID 03402)). The target values for the reduced annual precipitation were converted to monthly estimates. To calculate the actual required reduction, the reduced precipitation input at the precipitation manipulation subplots of the current month was compared with the target value. If the precipitation input was above or below the target value, the planned reduction and thus the tile coverage for the next month was chosen higher or lower according to the magnitude of deviation. For details of the roof construction and rainfall reductions see Baudis *et al*. [48].

**Table 1:** General plot information and characteristics of the understorey plant community.

Plot ID^a^	Exploratory	Main tree	Management type	Position		Soil type^b^	LAIsp		Species richness		H’		
				longitude	latitude		control	roof	control	roof		control	roof
Sbu	Schorfheide	beech	unmanaged	1.384.500	5.305.127	Cambisol	no veg.	no veg.	no veg.	no veg.	no veg.		no veg.
Sbm	Schorfheide	beech	managed	1.389.123	5.288.526	Cambisol	0.63 ± 0.18	0.88 ± 0.20	8	7	0.98 ± 0.32		0.85 ± 0.45
Scm	Schorfheide	pine (conifer)	intensively managed	1.386.399	5.307.658	Cambisol	0.62 ± 0.10	0.44 ± 0.13	5	3	0.87 ±0.23		0.76 ± 0.24
Hbu	Hainich	beech	unmanaged	1.045.518	5.110.069	Luvisol	0.40 ± 0.08	0.24 ± 0.09	3	3	0.63 ± 0.24		0.61 ± 0.12
Hbm	Hainich	beech	managed	1.037.834	5.117.893	Stagnosol	0.37 ± 0.04	0.42 ± 0.04	6	8	1.04 ± 0.33		1.46 ± 0.22
Hcm	Hainich	spruce (conifer)	intensively managed	1.031.074	5.127.165	Luvisol	0.92 ± 0.15	0.53 ± 0.09	5	5	0.96 ± 0.16		0.88 ± 0.22
Abu	Schwäbische Alb	beech	unmanaged	938.238	4.838.259	Cambisol	0.75 ± 0.21	0.44 ± 0.24	5	4	1.13 ±0.23		0.83 ±0.26
Abm	Schwäbische Alb	beech	managed	935.661	4.838.366	Leptosol	0.97 ± 0.05	0.71 ± 0.06	9	8	1.53 ±0.22		1.47 ± 0.19
Acm	Schwäbische Alb	spruce (conifer)	intensively managed	939.868	4.837.891	Cambisol	1.74 ± 0.41	1.63 ± 0.50	9	8	1.18 ± 0.20		1.42 ± 0.19

Plot and understorey properties were obtained from the control and precipitation manipulated subplots for the different exploratories and management types.

Species richness = total number of species found on the quadratic area of 2.45 m^2^, LAI_sp_ = leaf area index and H’ = Shannon’s diversity index; mean values and standard deviation of means are provided (n = 4). Plot Sbu had no understorey vegetation.

^a^ S = Schorfheide-Chorin; H = Hainich-Dün; A = Schwäbische Alb; bu = beech, unmanaged; bm = beech, managed; cm = conifer, intensively managed;

^b^ According to WRB [49].

### Understorey parameters

To assess the effect of understorey parameters on the bacterial community structure and to clarify if effects of forest management and reduced precipitation on bacterial community were modulated by the forest understorey, we measured leaf area index, plant species diversity (Shannon’s diversity index H’) and plant species richness of control and precipitation manipulated subplots after six months of precipitation reduction. For the subplot-specific leaf area index (*LAI*_*sp*_), we randomly collected field-fresh leaves from all species with a coverage > 5% at all subplots (about 1 g fresh weight per leaf sample which equals 2–12 leaves/species). We took digital photos in the field of leaves sorted by species. From the photos, specific leaf area (defined as the area of an average leaf of a given species) was determined using the image analysis program ImageJ 1.45s [50]. We also took digital photos of four randomly chosen quadratic areas per subplot (n = 4; total area = 2.45 m^2^) and counted the total number of leaves of each species within the known ground‐surface area and we calculated *LAI*_*sp*_ from these parameters. We also used the photos to quantify plant understorey diversity. We calculated the coverage (%) per species on the digital photos of the 2.45 m^2^ areas for each treatment to calculate the Shannon diversity index as described in Krebs [51].

### Soil sampling

The soil sampling campaigns took place in September 2012 (after six months of precipitation reduction). Mixed samples of about 200 g soil were taken from A_h_ horizons with a soil core sampler (0–5 cm), from each of the four precipitation manipulation and the corresponding control subplots. All samples were collected at a distance of 4–6 m from the adjacent tree. For the analyses of the bacterial community, an aliquot of 5 g per sample was immediately frozen and later transported on dry ice to the laboratory and stored there at -80°C. For the analyses of soil characteristics, the remaining soil samples were air dried and sieved to < 2 mm.

### Soil physical-chemical properties

To assess the effect of soil properties on the bacterial community structure and to clarify if effects of forest management and reduced precipitation on bacterial community were modulated by soil physical-chemical properties, we determined plot-specific soil conditions. Soil organic carbon (C_org_) and total nitrogen (N_t_) contents were determined by combustion using a LECO C/N analyser (Leco Corporation, St. Joseph, USA) according to DIN ISO 10694 [52]. Soil pH was measured in a supernatant of a soil suspension using 1:2.5 mixtures of soil and 10 mM CaCl_2_. We used soil texture data from the coordinated soil sampling campaign 2011 at the Biodiversity Exploratories; BExIS data base [47].

The development of the absolute soil water content of the upper 20 cm during the treatment and the difference between treatments and controls were calculated using the forest-hydrological model LWF- Brook90 [53]. LWF-Brook90 simulates the daily soil water budget as the result of infiltrating precipitation, water flow through the soil and water loss by evapotranspiration. LWF-Brook90 follows the approach of Shuttleworth and Wallace [54] to partition the total evapotranspiration into transpiration, soil evaporation, snow evaporation and interception evaporation. The following input data are required in daily resolution: precipitation, maximum and minimum air temperature, global radiation, vapour pressure, wind velocity. The necessary climate data were obtained for the period 2010 to 2013 from near-by stations of the German weather service (DWD station Angermünde (Schorfheide), Mühlhausen (Hainich), Münsingen- Apfelstetten (Schwäbische Alb)). The soil characteristics at the plots were available from soil profile descriptions (soil genetic horizons and their soil texture, bulk density, stone content). The water retention curve and the hydraulic conductivity of the soil horizons were estimated using a pedotransfer function [55]. Important model parameters which describe the effect of the vegetation on the local water budget were either obtained from field observations (depth distribution of roots), the BExIS database (id17687 forestEP stand structure and composition, stand density and tree age), approximated from literature (e.g. annual course of leaf area index), or set at values following the suggestions of the model developers. For the Pearson product-moment correlations, which were calculated between the NMS scores and soil water content, we computed the six month average of the absolute water content values for all 18 subplots.

### DNA and RNA extraction

Total DNA and RNA were co-extracted from 0.25 g soil (A_h_ horizon) following a modified protocol from Towe et al. [56]. Briefly, mechanical cell lysis using NucleoSpin Bead tubes (Macherey-Nagel, Germany) and the FastPrep-24 Instrument (MP Biomedicals, Germany; 40 s at 5.5 m s^-1^) was combined with a phenol-chloroform extraction followed by a purification using an AllPrep DNA/RNA Mini Kit (Qiagen, Germany). The standard protocol was amended by an on-column DNA digestion step applying RNase-Free DNase Set (Qiagen). The high content of humic acids of the forest soil required an additional purification of the RNA by an RNeasy MinElute Cleanup Kit (Qiagen). RNA was checked for contaminating DNA by PCR amplification with primers 8f and Eub518 targeting the 16S rRNA gene [57] and subsequently transcribed with the GoScript Reverse Transcription System (Promega, Germany).

### T-RFLP analysis and amplicon pyrosequencing

T-RFLP (Terminal Restrictions Fragment Length Polymorphism) analysis of the 16S rRNA gene was applied to analyse the metabolically active (RNA-based) as well as the total soil bacterial communities (DNA-based) from the nine plots with four replicates each for the control and the precipitation manipulation treatment. T-RFLP analysis was performed from DNA and cDNA by amplification of 16S rRNA gene fragments using the primers 8f (labelled with 6-FAM) and 926r followed by a digestion of the amplicons with the restriction enzyme *Hha*I [21]. Restriction fragments from two independent PCR reactions were pooled, mixed with 0.2μl ROX-labelled MapMarker 1000 (BioVentures, Murfreesboro, USA) and subsequently separated on an ABI 310 DNA sequencer (Applied Biosystems, Germany).

Based on the results of T-RFLP analysis, a deeper analysis of the metabolically active bacterial community was performed by amplicon pyrosequencing. For this purpose, cDNA samples were amplified with primers 8f and Eub518 targeting the V1-V3 region of the bacterial 16S rRNA gene. At their 5′ ends, the primers carried either the 454-adaptor A or the 454-adaptor B with a specific 6–7 nt barcode and a 2 nt linker for each soil sample (S1 Table). The barcodes differ in at least 2 nt and were selected from those applied by Schloss et al. [58]. Two independent PCR reactions were performed using AccuPrime Taq High Fidelity (Invitrogen). Cycling conditions were an initial denaturation of 1 min at 94°C, followed by 25 cycles of 20 s at 94°C, 30 s at 56°C and 5 min at 72°C. Combined amplicons were purified with the MSB Spin PCRapace kit (Invitek), quantified using a NanoDrop ND1000 spectrophotometer (Thermo Scientific) and pooled to get a mixed sample with equimolar amounts of all PCR products. Pyrosequencing was carried out on a GS FLX machine (Roche, Mannheim, Germany) using titanium reagents (GATC, Konstanz, Germany).

### Data processing and statistical analysis

Soil, understorey and diversity parameters of the microbial community were analysed for significant differences using a nested design. The differences between the three exploratories, the three management intensities and the reduced precipitation treatment *vs*. control were tested with a linear mixed effects model with exploratory, management and reduced precipitation treatment as fixed factors and subplot nested in plot as random factors. We consider the different management types as true replicates at the region level. Thus, each exploratory had experiments set up in different types of forest. As there were no replicates for the combination of exploratory and management type, we could not test for the interactions of both these factors. For the multiple comparisons of means we used the Tukey contrasts and corrected the p values using the Bonferroni method. The statistical analysis was carried out with R software (R-3.0.2, The R Foundation for Statistical Computing 2013; packages: lme4; nlme; [59, 60]). Significance of correlation between understorey parameters, soil physical-chemical properties, bacterial diversity parameters and MRPP A values were tested by Kendall's rank-based measure of association (R-3.0.2).

Terminal restriction fragments (T-RFs) with a size between 50 and 800 bp were determined using GeneMapper Software v. 4.0 (Life Technologies, Germany). T-RFLP profiles were standardized in a similar manner as suggested by Dunbar et al. [61]. The fluorescence of each peak was adjusted to the smallest profile and only peaks above the threshold of 100 fluorescence units were considered.

Processing of raw sequences obtained from pyrosequencing was carried out in Mothur v. 1.30.2 [62]. Sequences were optimized by trimming off primer and barcode sequences (primer differences allowed, 2 bp, barcodes, 1 bp) and removing sequences less than 300 bp and those with a quality score below 30. To remove potential pyrosequencing noise, reads differing by less than 1% of total residues were grouped by single linkage pre-clustering [63]. High-quality reads were aligned using the SILVA database, and chimeras removed using the Uchime algorithm [64]. After calculation of a distance matrix, operational taxonomic units (OTU) were generated using a cutoff of 0.03 and 0.10. For phylogenetic identification, the sequences were compared to the RDP 16S rRNA training set 9 using a confidence threshold of 80%. To equalize the number of sequences per sample, each group of sequences was subsampled to the size of the smallest group. Sequence flowgrams were deposited in the NCBI Short Read Archive (SRP040783).

To study shifts of the bacterial community structure a nonmetric multidimensional scaling (NMS) analysis was applied. The relative proportion of T-RFs and the distribution of OTUs within each sample determined by pyrosequencing, respectively, was used as input for calculating NMS by PC-ORD v.6.08 [65]. The presented NMS analyses were performed using Bray-Curtis distance measure. Stress values were in the range of 7.2 to 11.3 indicating a reliable test performance. Pearson product-moment correlations were calculated between the NMS scores and physical-chemical soil properties of samples as well as the aboveground plant community structure. Parameters showing significant correlation with at least one NMS axis (p < 0.05) were included as vectors on the ordination plot. To identify significant differences in T-RFLP profiles as well as the pyrosequencing data between exploratories, plots and reduced precipitation treatment were tested by means of Multi-Response Permutation Procedure (MRPP), which evaluates the observed against the permuted within-group agreement, applying rank transformation on proportional T-RF or OTU abundance [66]. MRPP reports a change-corrected index of within-group agreement (A), which increases with decreasing variability within the test groups as compared to differences between groups.

## Results

### Precipitation reduction and moderate drought

**Fig 1:**
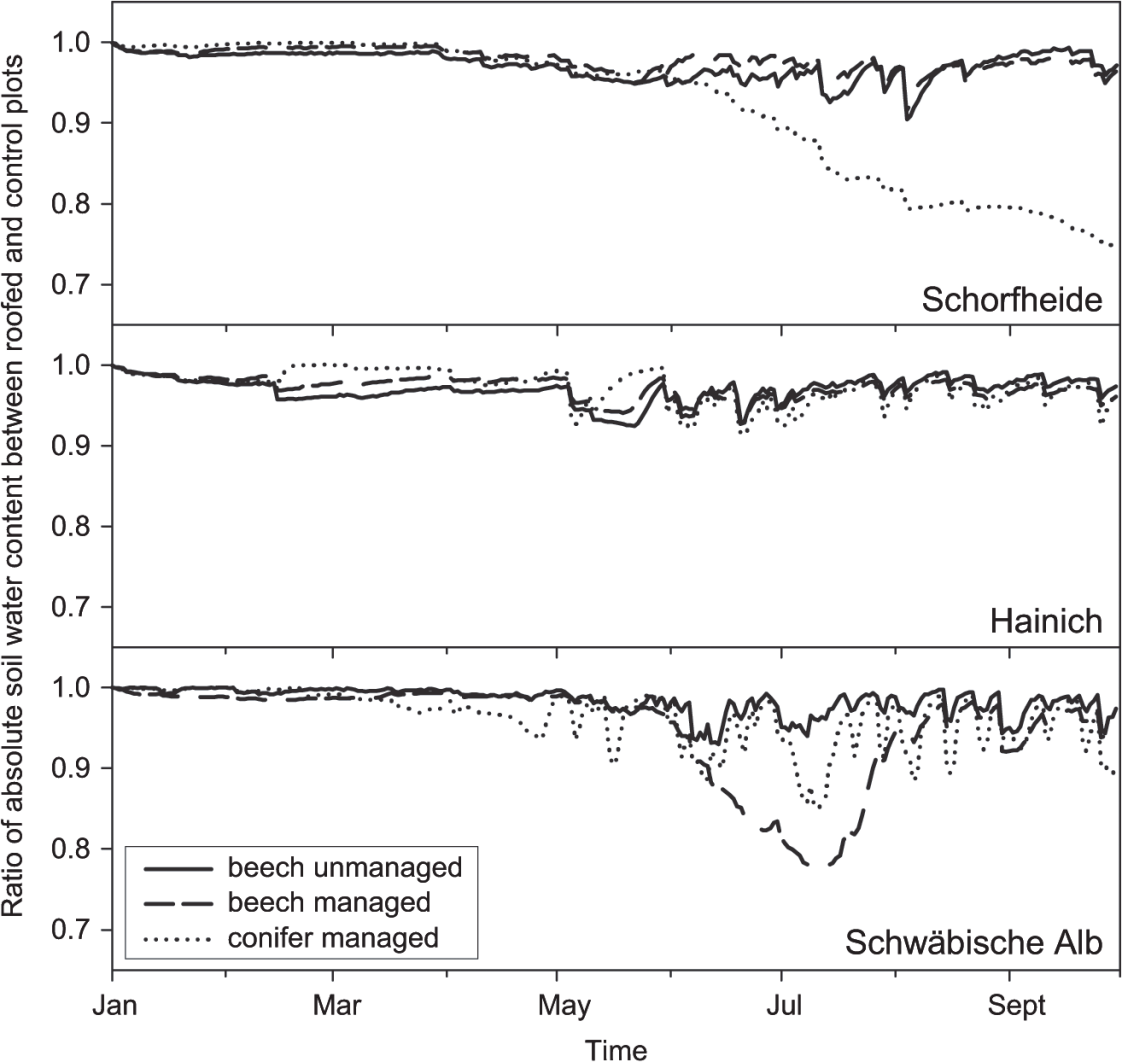
Ratio of the absolute soil water content between reduced precipitation and control subplots. The absolute water content in the upper 20 cm of the soil was estimated using the forest-hydrological model LWF- Brook90 for all three exploratories (S = Schorfheide; H = Hainich; A = Schwäbische Alb).

We simulated a statistically rare climate period by reducing precipitation over six months, resulting in a moderate drought in forest soils. Our precipitation reduction target level of the 2.5% percentile of cumulative annual precipitation corresponds to an average reduction of the incoming precipitation by 27% at Schorfheide, 33% at Hainich and 26% at the Schwäbische Alb. Until September 2012, precipitation reduction amounted to 109 mm at Schorfheide exploratory (reduction of incoming precipitation by 37.3%), 121 mm at Hainich (42.4%) and 126 mm at the Schwäbische Alb (20.9%). As indicated by the forest-hydrological model LWF-Brook90, the plant-available soil water storage (matric potential range between -63 hPa and -15,000 hPa) in the top 20 cm of the soil in the precipitation manipulation subplots was reduced by 2 to 8% compared with the control plots. The trend of the soil water content during the precipitation reduction is shown as ratio between manipulated and control subplots in Fig 1. During the six months of reduced precipitation, the strongest reduction of soil moisture occurred on the intensively managed pine plot at the Schorfheide exploratory, which is due to the remarkable stony and sandy soil profile of this plot. Most of the other plots showed uniform patterns of moderate drought with lowest reduction on the unmanaged plots at the Schorfheide and Hainich exploratories.

### Soil physical-chemical properties and understorey parameters

Soil properties and understorey parameters were analysed to assess if the effect of reduced precipitation on the bacterial community structure might be modulated or overlaid by other drivers. Soil pH was lowest at the Schorfheide plots (S2 and S4 Tables, significantly different in comparison to other exploratories). The intensively managed conifer plots in each exploratory contained the most acidic soils (S2 Table). The Schwäbische Alb had significant higher N_t_ values compared to both other exploratories (S4 Table). N_t_ was lower at the precipitation manipulation subplots compared to the controls in the managed beech forests. All three soil parameters (pH, C_org_ and N_t_) showed significant interactions between the management and the treatment factor, but significant effects of the precipitation reduction as single factor could not be determined (S4 Table).

The species richness and Shannon’s diversity index of the understorey plant community were significantly higher at the Schwäbische Alb exploratory compared to the other two exploratories (Table 1 and S5 Table). Furthermore, the plant species richness was significantly lower at the unmanaged beech plots than at the other two management intensities. While there were significant lower LAI_sp_ values at the precipitation manipulation subplots compared to the controls, no significant differences were found for species richness and understorey diversity (S5 Table).

### Soil bacterial community structure analysed by T-RFLP

T-RFLP analysis of the 16S rRNA gene was applied to analyse the metabolically active as well as the total soil bacterial communities. Both the active and the total communities (RNA and DNA-based) displayed complex and reproducible T-RFLP profiles. The number of T-RFs in the individual profiles ranged from 36–71.

#### Total bacterial community

**Fig 2:**
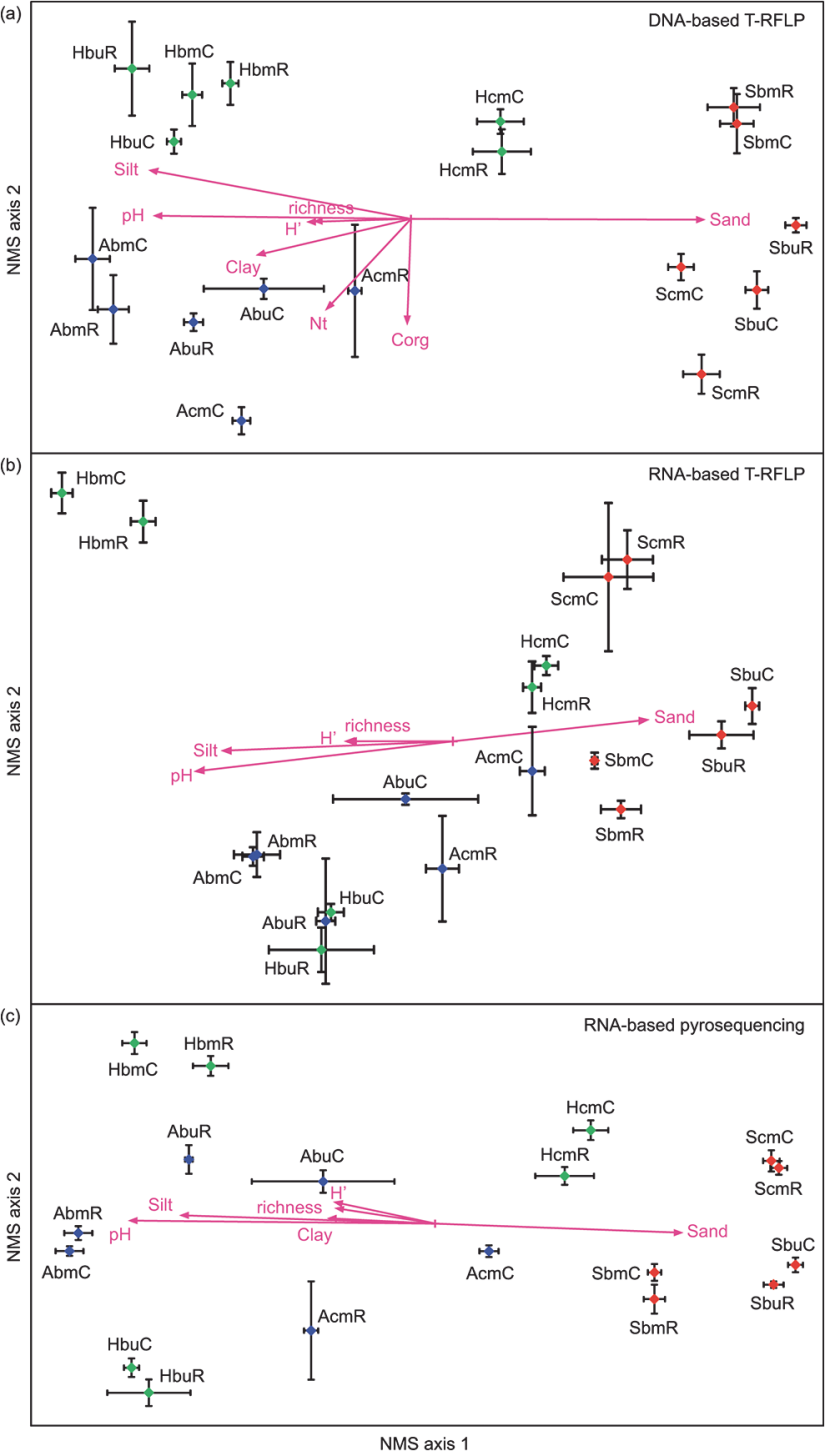
NMS ordination plots of the bacterial community structure obtained from reduced precipitation (R) and control (C) subplots. Soil samples were taken from nine plots of the three exploratories in September 2012. The community structure of the metabolically active (b, c) and the total (a) bacteria were analysed by T-RFLP (a, b) and tag-pyrosequencing (c). Statistically significant correlations (p < 0.05) of soil characteristics (C_org_: soil organic carbon; N_t_: total nitrogen) and understorey parameters (richness: species richness and H’: Shannon’s diversity index of the understorey plant community) were indicated by arrows. For plot ID see Table 1.

The DNA-based profiles revealed significant variation between the three exploratories and all studied plots as determined by the means of MRPP test (mean A = 0.38; p < 0.0001, Fig 2A). The visualization of changes within the bacterial community structure using the NMS ordination plot showed a clear separation of the three exploratories and a strong plot-specific community structure (Fig 2A). Furthermore, some effects of the management intensity were obvious. At the Hainich exploratory, the spruce dominated intensively managed plot clustered separately from the beech-dominated plots. The precipitation manipulation and the control subplots clustered together in most cases. Only two plots displayed a significant difference between the treatments (Fig 2A and Table 2). At the Schwäbische Alb, the highest heterogeneity as well as the highest effect of reduced precipitation was observed in the spruce plot. The soil characteristics and understorey parameters were found to significantly correlate with the first or second ordinate axis (p < 0.05; Fig 2A). Soil pH and texture mainly correlated with the first axis (mean r^2^ = 0.63) corresponding to the separation between the Schorfheide plots on the one hand and the Hainich/ Schwäbische Alb plots on the other. In contrast, correlation of C_org_ and N_t_ with the second axis (mean r^2^ = 0.27) matched the differentiation between Hainich and Schwäbische Alb. Species richness and diversity of the understorey plant community showed a mean r^2^ of 0.26 to the first axis indicating a weaker but significant correlation with the main separation of the bacterial community of the exploratories (Fig 2A).

**Table 2:** Significance test (MRPP) of the effect of reduced precipitation on the bacterial community structure.

Plot ID^a^	Management type	Total bacteria(DNA-based T-RFLP)	Active bacteria(RNA-based T-RFLP)	Active bacteria (RNA-based pyrosequencing)
Sbu	unmanaged	0.26*	0.21*	0.41**
Sbm	managed	-	0.33**	0.34**
Scm	intensively managed	-	-	0.32**
Hbu	unmanaged	-	0.19*	0.17*
Hbm	managed	-	0.22*	0.28**
Hcm	intensively managed	-	-	0.26**
Abu	unmanaged	-	0.22*	0.32*
Abm	managed	-	-	0.18*
Acm	intensively managed	0.30**	0.37**	0.36**

^a^ Pairwise comparison of the control and precipitation manipulated subplots. For plot ID see Table 1;

* p < 0.05,

** p < 0.01

#### Metabolically active bacterial community

The MRPP test of the RNA-based T-RFLPs indicated clear differences in the active bacterial community between the Schorfheide and the Schwäbische Alb (A = 0.34, p < 0.0001; Fig 2B). As demonstrated in the NMS plot, the Hainich exploratory showed a high level of heterogeneity between the plots and was not clearly separated from the other two exploratories. The Hainich beech unmanaged plot clustered separately from the other plots. Interestingly, the three intensively managed plots planted with conifers clustered adjacent to each other (significant different from managed and unmanaged plots with a mean A = 0.16, p < 0.0001; MRPP; cf. Fig 2B). As already shown for the DNA-based profiles, the community structure of the active bacteria was highly plot-specific (mean A = 0.44, p < 0.0003; MRPP). At six of the nine plots, a significant effect of the reduced precipitation treatment on the RNA-based T-RFLP patterns could be detected. The highest precipitation manipulation effect was observed in the spruce plot at the Schwäbische Alb and in the beech managed plot at the Schorfheide (Table 2). The effect of soil properties was less pronounced on the active bacterial community compared to the impact on the total community (DNA-based). Merely, soil pH, silt and sand showed a significant correlation with the first axis. The understorey diversity parameters displayed a comparable correlation with the first axis (r^2^ = 0.28) indicating a significant association of the plant community and the community structure of the active soil bacteria (Fig 2B).

### Metabolically active bacterial community studied by pyrosequencing

Based on the results of the T-RFLP analysis, we studied the metabolically active bacterial community in more detail to reveal information about the specific changes within the community as well as to increase resolution of the community analysis. We analysed the 16S rRNA gene amplicons of the V1-V3 region by tag-pyrosequencing prepared from the RNA that originated from the nine plots with four replicates each of the control and reduced precipitation treatment. In total, 628,010 non-chimeric, high quality reads with a median sequence length of 460 nucleotides were obtained. All groups of sequences were subsampled to 3,933 reads each, which was the size of the smallest sample (S1 Table). The sequences were used to form OTUs at genetic distances of 0.03 and 0.10, corresponding to pairwise similarities of above 97% and 90%, respectively for all of the sequences in an OTU. Total numbers of OTUs in the data set were 17,447 and 3,563, respectively. Per sample, the number of OTUs ranged from 766 to 1618 and 222 to 576 (Table 3 and S1 Table). Based on OTUs of 97 and 90% similarity, mean Good’s coverages of 0.82 and 0.95 were achieved, suggesting that the data were not sufficient to capture the final richness even at a genetic distance of 0.10.

**Table 3:** Coverage and diversity of OTUs for each of the subplots as identified by RNA-based pyrosequencing.

Plot ID^a^	Management type	Treatment	OTU richness	Coverage	H	Inverse Simpson index (1/D)
Sbu	unmanaged	Control	905	0.87	5.9	96.2
		Roof	1024	0.85	6.13	124.9
Sbm	managed	Control	1288	0.79	6.47	145.8
		Roof	1293	0.8	6.52	155.1
Scm	intensively managed	Control	856	0.88	5.82	102.6
		Roof	779	0.9	5.7	97.4
Hbu	unmanaged	Control	1448	0.77	6.73	154.2
		Roof	1382	0.78	6.6	146.1
Hbm	managed	Control	1238	0.8	6.21	71.5
		Roof	1235	0.8	6.28	85.6
Hcm	intensively managed	Control	910	0.87	5.86	90.4
		Roof	1092	0.84	6.16	112.8
Abu	unmanaged	Control	1523	0.76	6.88	238.5
		Roof	1554	0.75	6.91	262.8
Abm	managed	Control	1485	0.77	6.89	300.5
		Roof	1520	0.77	6.92	283.8
Acm	intensively managed	Control	1117	0.84	6.23	127.2
		Roof	1317	0.8	6.7	226.1

Mean values of the four replicates are provided. The data are based on an OTU distance of 0.03. OTU richness = number of OTUs; H: non-parametric estimate of the classical Shannon’s diversity index.

^a^ For plot ID see Table 1.

Despite this restriction, OTU richness showed significant differences between the three exploratories. Moreover, inverse Simpson (1/D) and non-parametric Shannon indexes were significantly increased at the Schwäbische Alb (Table 3 and S6 Table; based on OTUs of 97% similarity). Moreover, significant differences could be found between the three management intensities for Shannon index and OTU richness. Differences in bacterial diversity and OTU richness between the precipitation manipulation and control subplots could not be determined, but we detected a significant interaction between the management intensity and the precipitation reduction for the OTU richness (S6 Table). The multiple comparisons indicated that the OTU richness was significantly different for the precipitation reduction subplots compared to the controls just for the intensively managed conifer plots (Table 3 and S6 Table).

The OTU-based approach was used to calculate NMS using Bray-Curtis dissimilarity which does not overemphasize the variance of low-abundant OTUs. As a result, the ordination plot showed a clear differentiation of the community structure between the studied plots (Fig 2C). In comparison to the T-RFLP analysis, significantly reduced within-plot variability was observed. Apart from that, the pyrosequencing data showed a similar pattern as compared to the RNA-based T-RFLPs. The Schorfheide plots were distinguished from the other two exploratories which was found to be significant after MRPP (mean A = 0.39, p < 0.0001) and the community structure of all studied plots were significantly different (mean A = 0.45, p < 0.0003). The intensively managed plot again clustered separately from the beech dominated plots at Hainich (Fig 2C). As a further effect of management—though less obvious than in the RNA-based T-RFLP—the three intensively managed conifer plots were grouped together (significantly different from managed and unmanaged beech plots with a mean A = 0.16, p < 0.0003, MRPP). Based on the reduced variability of the subplots, a significant effect of the reduced precipitation could be revealed for all studied plots (Table 2). Correlations with soil characteristics and understorey diversity parameters were comparable with the RNA-based T-RFLP as well. Soil pH and texture showed a mean correlation of r^2^ = 0.59 with the first ordinate axis, mean r^2^ of understorey richness and diversity was 0.26. The six month average of the absolute soil water moisture values did not significantly correlate with any of axes for all three NMS plots indicating that other (soil) parameters were stronger drivers of the bacterial community structure.

**Fig 3:**
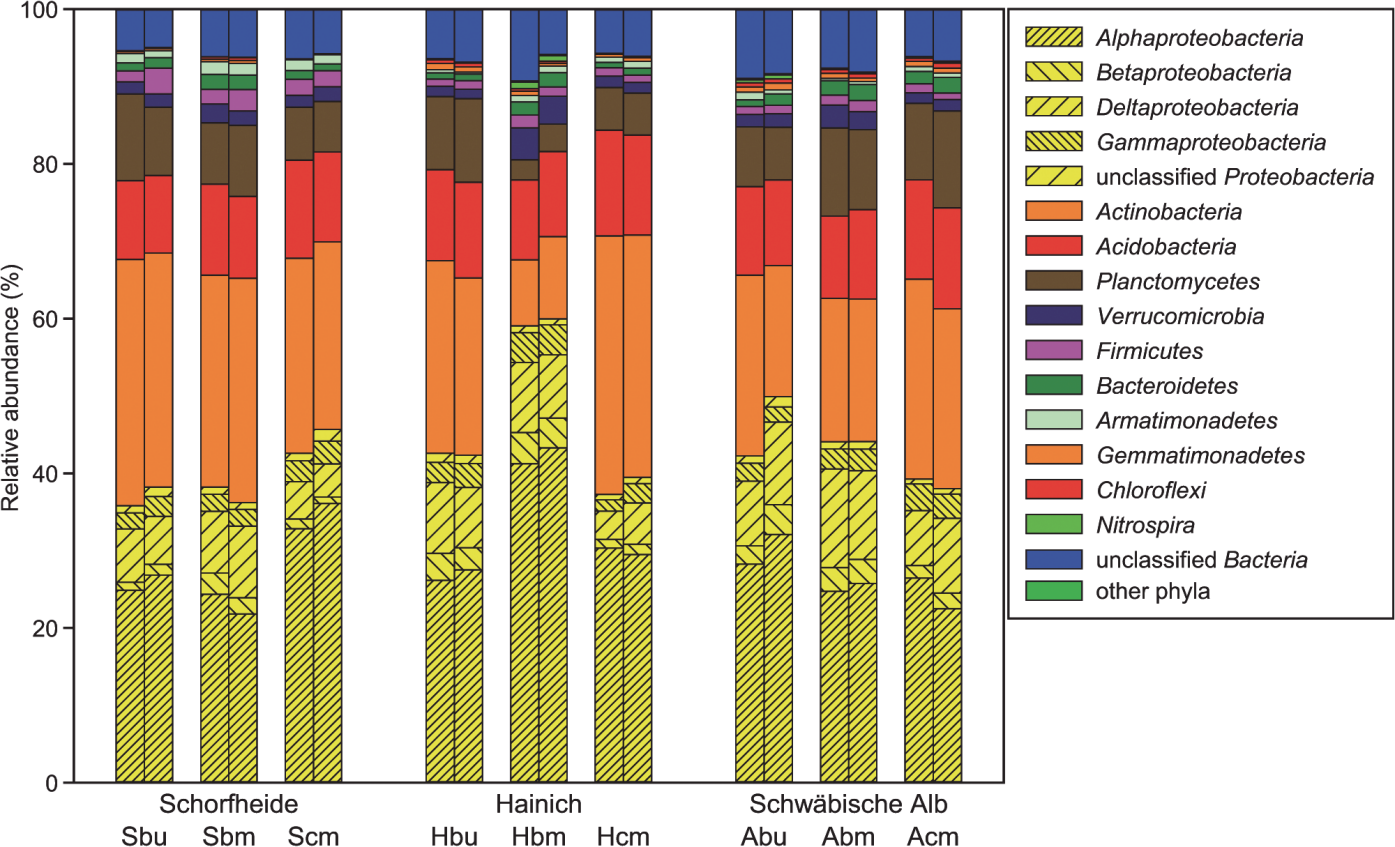
Distribution of bacterial phyla across the different plots and treatments. The main phylum (*Proteobacteria*) is subdivided in classes. For plot ID see Table 1, left bar—control, right bar—reduced precipitation subplot.

Analysis of the phylotypes obtained from the pyrosequencing data revealed the presence of 11–15 phyla per plot with highest number of phyla at the exploratory Schwäbische Alb (S3 Table). The data revealed a dominance of the phyla *Proteobacteria* (42.9%), *Actinobacteria* (23.7%), *Acidobacteria* (11.6%) and *Planctomycetes* (8.1%). Differences in relative abundance of some groups were visible between the exploratories (a lower abundance of *Proteobacteria* at Schorfheide and of *Planctomycetes* at Hainich as well as a higher abundance of *Deltaproteobacteria* at Schwäbische Alb (Fig 3). Management intensities also influenced the relative abundances of bacterial phyla and proteobacterial classes. So, intensively managed conifer plots harboured less *Deltaproteobacteria* and more *Acidobacteria* whereas managed beech plots showed a higher abundance of *Actinobacteria*. A clear plot-specificity of the distribution of phyla and proteobacterial classes was found for the Hainich beech managed plot, which differed in the relative proportion of *Alphaproteobacteria*, *Actinobacteria* and *Planctomycetes* from the other Hainich plots as well as from the Schorfheide and Schwäbische Alb exploratories.

**Fig 4:**
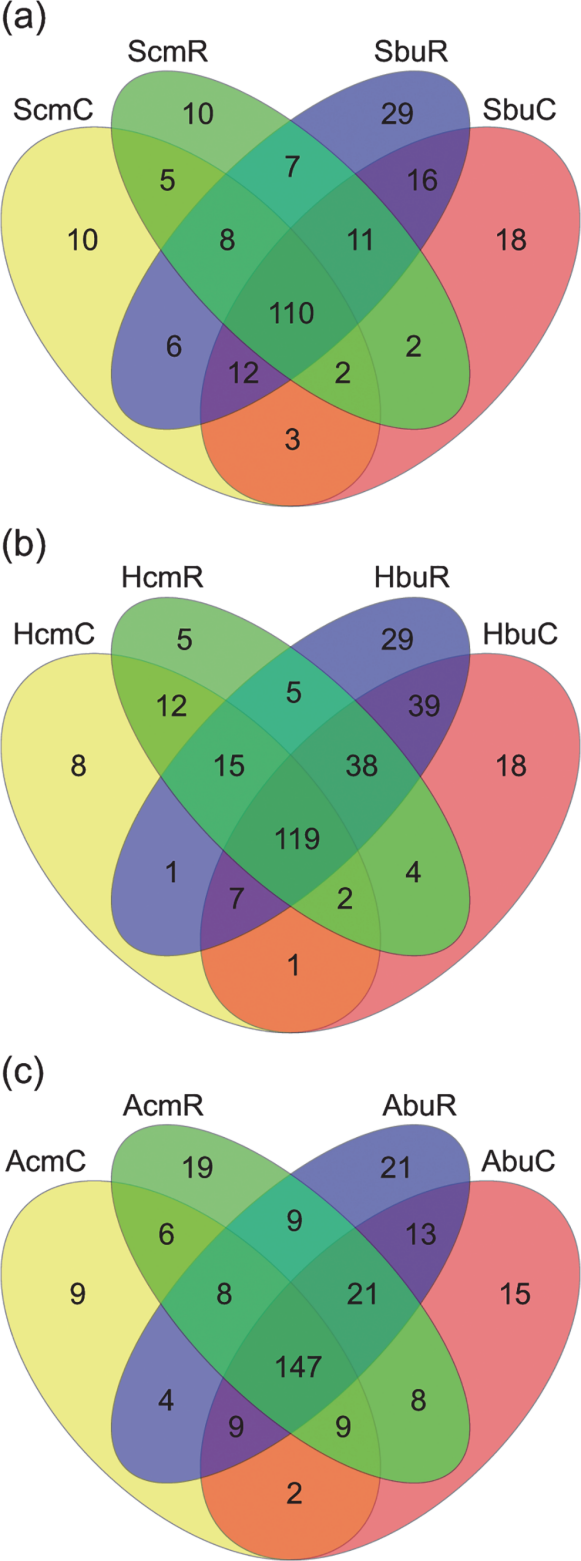
Differences in the number of phylotypes at the genus level. The bacterial community was compared between the reduced precipitation (R) and the control (C) subplots of conifer intensive (left) and beech unmanaged (right) plots. (a—Schorfheide, b—Hainich, c—Schwäbische Alb). For plot ID see Table 1.

At the genus level, the number of phylotypes increased with the understorey vegetation diversity and understorey species richness (τ = 0.25; p = 0.002; τ = 0.25; p = 0.004, respectively, S3 Table). These results were in accordance with the significant association of plant community and bacterial community structure shown in the NMS (see above). We detected significantly less phylotypes at the Schorfheide exploratory compared to the other exploratories (S3 and S6 Tables). The conifer plots demonstrated at all taxonomic levels fewer phylotype numbers (S3 Table). For the number of phylotypes we found a significant effect of the reduced precipitation treatment and a significant interaction between the two factors treatment and management. The multiple comparisons indicated that the precipitation reduction subplots had significantly more phylotypes at the genus level than the control subplots at the intensively managed plots (Fig 4 and S6 Table).

As shown in the venn diagrams in Fig 4, the majority of phylotypes found at the unmanaged beech plots of the three exploratories were also identified at the respective intensively managed conifer-planted plots. However, the conifer plots harboured not only considerably less specific phylotypes but also shared less phylotypes between the control and reduced precipitation subplots, both indicating a lower richness at the genus level (cf. S3 Table). The venn diagrams also illustrate for the Schorfheide exploratory that there was a considerable number of phylotypes present only in the precipitation reduction or in the control treatment (Fig 4A). At Hainich and Schwäbische Alb, similar proportions of control and reduced precipitation specific phylotypes were observed (Fig 4B and 4C). With the exception of the intensively managed plot at the Schorfheide exploratory, the precipitation manipulation subplots had more phylotypes at the genus level than the control subplots (Fig 4).

**Fig 5:**
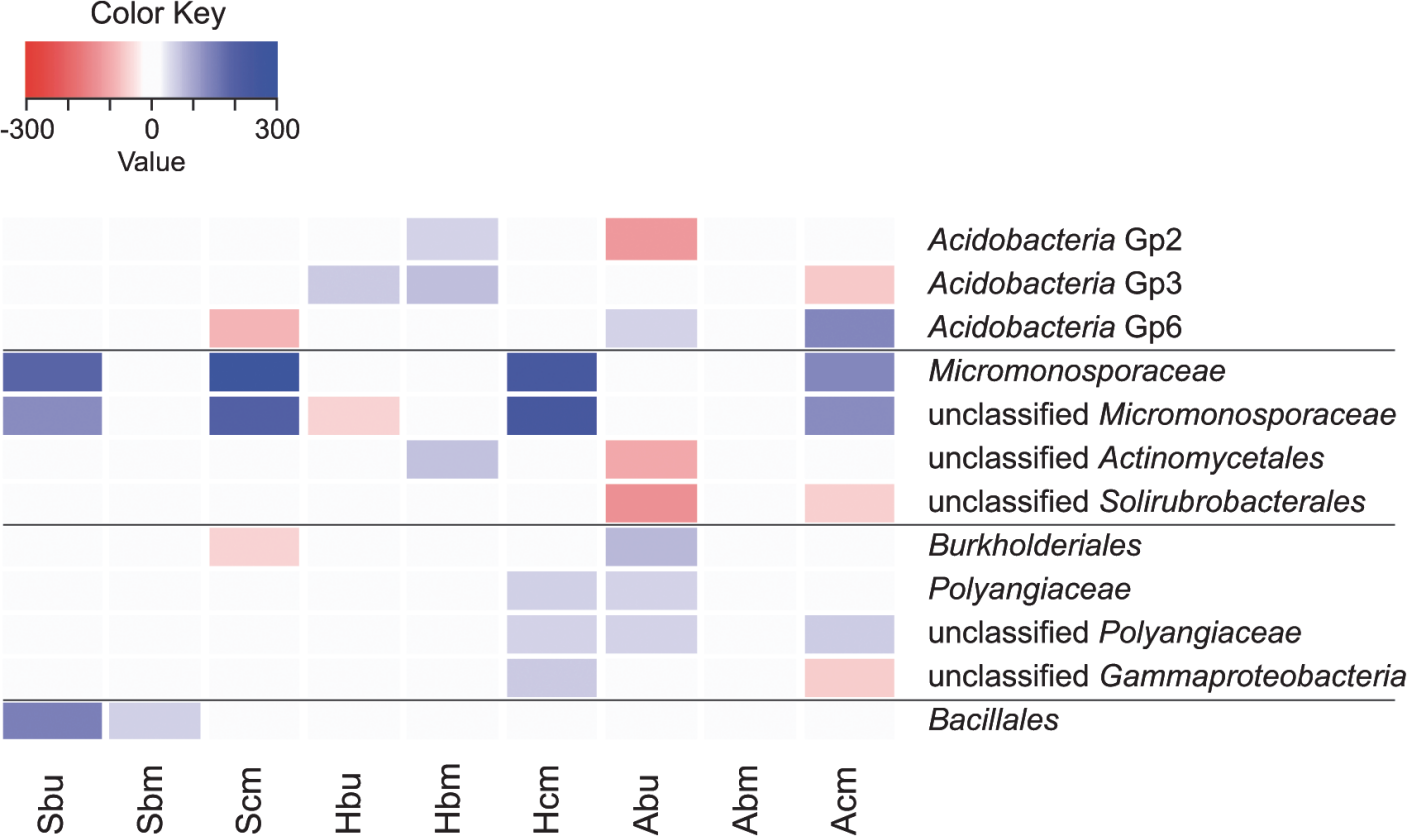
Phylotypes with abundance shifts between the reduced precipitation and the control subplots. Shown groups had a relative abundance of more than 1% of the bacterial community and increased or decreased by more than 50% due to reduced precipitation. For plot ID see Table 1.

The identified phylotypes were analysed to reveal taxonomic groups which intensively responded to the reduced precipitation treatment. Comparison of manipulated and control subplots revealed 12 phylotypes with a relative abundance of more than 1% of the bacterial community that were increased or decreased by more than 50% due to reduced precipitation treatment on at least two of the nine studied plots (Fig 5). Four of the affected phylotypes belonged to the phylum *Actinobacteria;* the others were classified as *Proteobacteria*, *Acidobacteria or Firmicutes*. The most impacted groups are within the family *Micromonosporaceae*, which increased in relative abundance by up to 300% as a result of precipitation reduction.

In our search for plot-specific characteristics, which might modify the overall resistance of bacterial communities to reduced precipitation, we correlated the A values of the MRPP (an indicator of the effect strength of the reduced precipitation on the active bacterial community, Table 2) against ratios between reduced precipitation and control subplots of understorey vegetation parameters, physical-chemical soil parameters and soil water content (Table 1, S2 Table and Fig 1). We did not find a significant relationship between changes in these parameters and the intensity of the shift in the bacterial community structure upon precipitation reduction.

## Discussion

We imposed a precipitation reduction—resulting in moderate reduction of soil moisture—on the forest understorey to investigate putative shifts in total and active bacterial communities. We carried out the work at three different Biodiversity Exploratories across Germany with three different plots along a management intensity gradient at each exploratory.

### Bacterial community structure of the studied forest soils

Since soil microorganisms are thought to be in large part inactive [67] and DNA can persist in dead cells and as extracellular DNA in soils [68], DNA based approaches do not necessarily reflect the microbial groups currently performing metabolic functions. RNA-based methods have been increasingly used to analyse metabolically active members of the microbial communities. The amount of rRNA per cell roughly correlates with the growth activity of bacteria [69] and allows the detection of living microorganisms constituting most of the metabolic activity. Though this approach also has pitfalls, such as the extraction of RNA from soil [70], varying ribosome contents per cell and the occurrence of RNA reserves in dormant cells [71, 72], RNA based surveys represent a suitable strategy for focussing on metabolically active microbial communities.

In this study, the analysis of the active bacterial community by RNA-based pyrosequencing revealed a dominance of *Proteobacteria* (mainly *Alpha*- and *Deltaprotebacteria*), *Actinobacteria*, *Acidobacteria* and *Planctomycetes* across all plots investigated (Fig 3), which is mostly in agreement with other studies on forest soils [22, 73, 74]. When we compare the community structure of total bacteria versus active bacteria studied in this paper, *Acidobacteria* were obviously less abundant while a higher proportion of *Actinobacteria* and a significant proportion of *Planctomycetes* were observed (Fig 3) [73, 74]. Studies analysing the active bacterial community (RNA-based) of forest soils are rare. For a *Picea abies* forest, Baldrian et al. [75] similarly found an increased proportion of *Actinobacteria* in the active as compared to the total soil bacterial community. In grassland soils, a comparison of total and active bacteria demonstrated a distinct shift from *Acidobacteria* to *Actinobacteria* and the occurrence of *Planctomycetes* within the active bacteria [16], both supporting our finding.

### Exploratory and management effects on the bacterial community structure

We wanted to examine whether the total and active microbial community structure varies between the three exploratories due to different soil properties and management intensities. We found the bacterial community structure was most influenced by soil characteristics (Fig 2). This was exemplified by the divergence of Schorfheide in community structure from the other exploratories. The soils here had significantly lower pH values and the textural properties (i.e., sand and stone content) were comparatively different from the other two exploratories. The dominance of pH and soil texture as drivers of the bacterial community structure in forest soils is an agreement with recent studies [22, 76, 77. Soil texture might regulate bacterial colonization and distribution through effects on habitat heterogeneity, competition between bacteria and fungi and/or protozoan grazing [17, 78, 79].

Beyond these exploratory specific effects, there was an effect of overstorey tree species on the bacterial dynamics. We found that the active bacterial communities associated with conifer species (clearly visible in the RNA-based T-RFLPs and with a tendency in the pyrosequencing data) were more similar to each other across the different exploratories than to the beech managed or unmanaged community within a given exploratory (Fig 2B and 2C). Furthermore, fewer phylotypes and OTUs as well as the lowest diversity were observed at the intensively managed conifer plots across all exploratories (Table 3 and S3 Table). We also found a higher abundance of *Acidobacetria* and lower abundance of *Deltaprotebacteria* at these plots (Fig 3). Our findings extend the results of Nacke et al. [74] who studied the total soil bacterial community of the exploratory Schwäbische Alb to the active bacterial community at forests in different regions across Germany. They concluded for the exploratory Schwäbische Alb that harvesting type (beech age class forest or unmanaged beech forest) has a minor or no impact on soil bacterial community structure, which was largely driven by tree species. Additionally, we observed effects of management intensity on the understorey plant community, which correlated well with impacts on the active bacterial community structure and diversity (Fig 2, Table 3 and S3 Table). Wubet et al. [80] observed that the understorey vegetation affected the soil fungal community structure, which supports our finding that the bacterial community was indirectly influenced by the understorey plant community.

The results confirm our primary expectation as we found clear differences in both the total and active bacterial community structure between the three exploratories. We summarise that the main drivers for the total and active bacterial community structure were soil pH and texture, but overall the effect of soil characteristics on the active bacteria was less pronounced. Management intensity *per se* did not affect the bacterial community composition and thus we have to modify the second part of hypothesis and conclude that the main overstorey tree species—and thus the specific forest management favouring conifer replacement—together with the understorey plant community influences the soil microbial community structure with particular effects on the active bacteria.

### Reduced precipitation effect on bacterial community structure

We hypothesized that reduced precipitation would change the bacterial community. We could in fact detect effects of precipitation reduction and the concomitant decrease in soil moisture on the bacterial community structure. The effect was hardly distinguishable in the total bacterial community including the large part of the soil bacteria that are metabolically inactive [17], but we observed a stronger response to precipitation reduction in active and growing cells. Beside the shift found in the OTU-based approach, the reduced precipitation subplots often had more phylotypes at the genus level as compared to the subplots without precipitation reduction (Fig 5 and S3 Table). This indicates that some phylotypes might have become more active with reduced soil water content. Comparable studies also detected significant shifts in total bacterial community composition, i.e. under long-term drought treatments in a steppe [81], and after three and ten months of throughfall exclusion in a tropical forest [20]. Consistent to our findings, Barnard et al. [16] found that drought affected the relative abundance of active bacteria at phylum and class levels, but responses to changes in water availability were relatively small in most groups of the total (DNA-based) bacteria. In other long-term studies drought effects were coincident with further changes in soil properties and the bacterial community structure was potentially more strongly driven by other environmental factors that changed under long-term drought than by water deficiency directly [81]. In our study, however, soil properties such as pH, C_org_ or N_t_ were not affected by drought and as a consequence we infer that either direct effects of reduced soil moisture or changes in the plant-microbe interaction upon drought affected the active bacterial community.

To date, most investigations have focused on extreme drought events often followed by subsequent rewetting [18, 44]. There is, however, little information available on potential changes in microbial community under moderate and potentially more realistic drought over the growing season. A roof experiment in a spruce forest with a rainfall reduction for 11 months and a moderate volumetric water content reduction between 19–27% v/v indicated that drought had no clear effects on microbial biomass and activity. The authors attributed the high variability of results to distinct spatial variability of top soil properties as well as the rather low intensity of the experimental drought [82]. We could also show in our study that such moderate, though realistic droughts, which are expected to occur more frequently in Central Europe in the future, did not drastically affect the bacterial community. There were negligible impacts on the total community composition but more pronounced effects on the metabolically active community. However, the strength of this effect was rather low especially as compared to the other environmental impacts that control the soil bacterial community.

The search for phylotypes which were especially affected by precipitation reduction revealed some taxa belonging to *Proteobacteria*, *Acidobacteria*, *Firmicutes* and *Actinobacteria* (Figs 3 and 5). Bouskill et al. [83] observed an increase in the relative abundance of some members from the *Actinobacteria*, *Planctomycetes*, and *Proteobacteria* and a decrease in some *Acidobacteria*, *Bacteroidetes* and *Firmicutes* under precipitation throughfall exclusion. They concluded that the bacteria with higher relative abundance could be taxa that exhibit a greater tolerance towards osmotic stress and potentially a preference for elevated solute concentrations. Our results support this general partitioning, as we found that e.g. *Bacillales* had higher abundances and *Proteobacteria* groups showed only a weak response suggesting that in most cases the effect of precipitation reduction is group-specific far below the phylum level. However, it is noticeable that the most impacted groups in our study represent filamentous *Actinobacteria*. Filamentous (mycelium-forming) bacteria use this growth form to facilitate growth and expansion under conditions of low hydraulic connectivity (drought conditions) in unsaturated soils [84], which could be an explanation for stimulated growth under the moderate drought conditions induced here. Barnard et al. [16] also found a relative increase of active *Actinobacteria* and decrease of *Acidobacteria* with summer drought and concluded that these contrasting drought related changes in abundance may reflect different bacterial life-strategies. In our study, the groups sometimes responded with both a decrease or an increase of the relative abundance in different plots. This could point to differences in the resistance of the community dependent on the respective plot properties.

Finally we aimed at assessing, which plot specific characteristics as well as forest management intensities would affect the overall resistance of the microbial community structure to the precipitation reduction. We found several parameters (soil characteristics, main overstory tree species, understorey) that influenced the soil bacterial community. However, all these factors interact, forming a strong plot-specificity that is clearly indicated by the plot-specific clustering of the bacterial community structure (Fig 2). Keeping this strong impact in mind, we observed clear differences of the moderate drought effect for the nine studied plots and thus can confirm our hypothesis. The highest drought effect was found for the Schorfheide beech unmanaged plot and the Schwäbische Alb conifer managed plot where not only the active but also the total community was affected. On the other hand, the bacterial community of some plots, such as the Schorfheide conifer managed plot, only marginally responded to the precipitation reduction even though the relative reduction of soil moisture was highest on that plot. The resistance of soil microbial communities to environmental stressors is not only determined by soil structure and substrate quality but also modified by the interaction with the vegetation [17]. In the case of our studied plots we found a differentiated response to the precipitation reduction and the resulting moderate drought but we were unable to further reveal single parameters modifying this resistance.

## Conclusion

Our results show that a rainfall reduction with a return interval of 40 years, which might occur more frequently in future [1], resulted in significant effects on the active soil bacterial community. However, the strength of the effect was rather low, especially in comparison to the strong evidence that both the different soil characteristics and the overstorey tree species together with the understorey vegetation, mainly drive the bacterial community structure. The effect of the moderate drought treatment during six months differed between the studied plots, but we could not identify specific parameters such as forest management or understorey plant community, that might modify the resistance of the bacterial community toward the moderate drought. The fact that a decrease in precipitation started to affect the active but not the total bacterial community points to an adequate resistance of the soil microbial system during drought periods occurring within a single growing season.

## Supporting Information

S1 Fig Rain shelters for precipitation reduction. Assembly of the roofs (a), construction of a single roof (b) and a view indicating the acrylic transparent tiles and the rain gutters (c) are shown. Pictures were taken at the unmanaged beech plot of the Schorfheide-Chorin exploratory.
(TIF)

S1 Table List of samples and barcodes. (DOCX)

S2 Table Physical-chemical soil properties for each of the subplots. (DOCX)

S3 Table Mean number of phylotypes at the five different taxonomic levels obtained for each of the subplots. (DOCX)

S4 Table Analysis of variance of the linear mixed effects models for the soil organic carbon content (C_org_), total nitrogen (N_t_) and pH. (DOCX)

S5 Table Analysis of variance of the linear mixed effects models for the understorey diversity parameters. (DOCX)

S6 Table Analysis of variance of the linear mixed effects models for the diversity parameters of the pyrosequencing data. (DOCX)
