# Correction: Soil Bacterial Community Structure Responses to Precipitation Reduction and Forest Management in Forest Ecosystems across Germany

**DOI:** 10.1371/journal.pone.0127608

**Published:** 2015-04-30

**Authors:** Katja Felsmann, Mathias Baudis, Katharina Gimbel, Zachary E. Kayler, Ruth Ellerbrock, Helge Bruehlheide, Johannes Bruckhoff, Erik Welk, Heike Puhlmann, Markus Weiler, Arthur Gessler, Andreas Ulrich

The sixth author's name is spelled incorrectly. The correct name is: Helge Bruelheide. The correct citation is: Felsmann K, Baudis M, Gimbel K, Kayler ZE, Ellerbrock R, Bruelheide H, et al. (2015) Soil Bacterial Community Structure Responses to Precipitation Reduction and Forest Management in Forest Ecosystems across Germany. PLoS ONE 10(4): e0122539. doi:10.1371/journal.pone.0122539
