# Supplementary figures and images for: Soil Bacterial Community Structure Responses to Precipitation Reduction and Forest Management in Forest Ecosystems across Germany
Source: PLoS One. 2015 Apr 14;10(4):e0122539. doi: 10.1371/journal.pone.0122539 (PMC4397059; doi:10.1371/journal.pone.0122539)

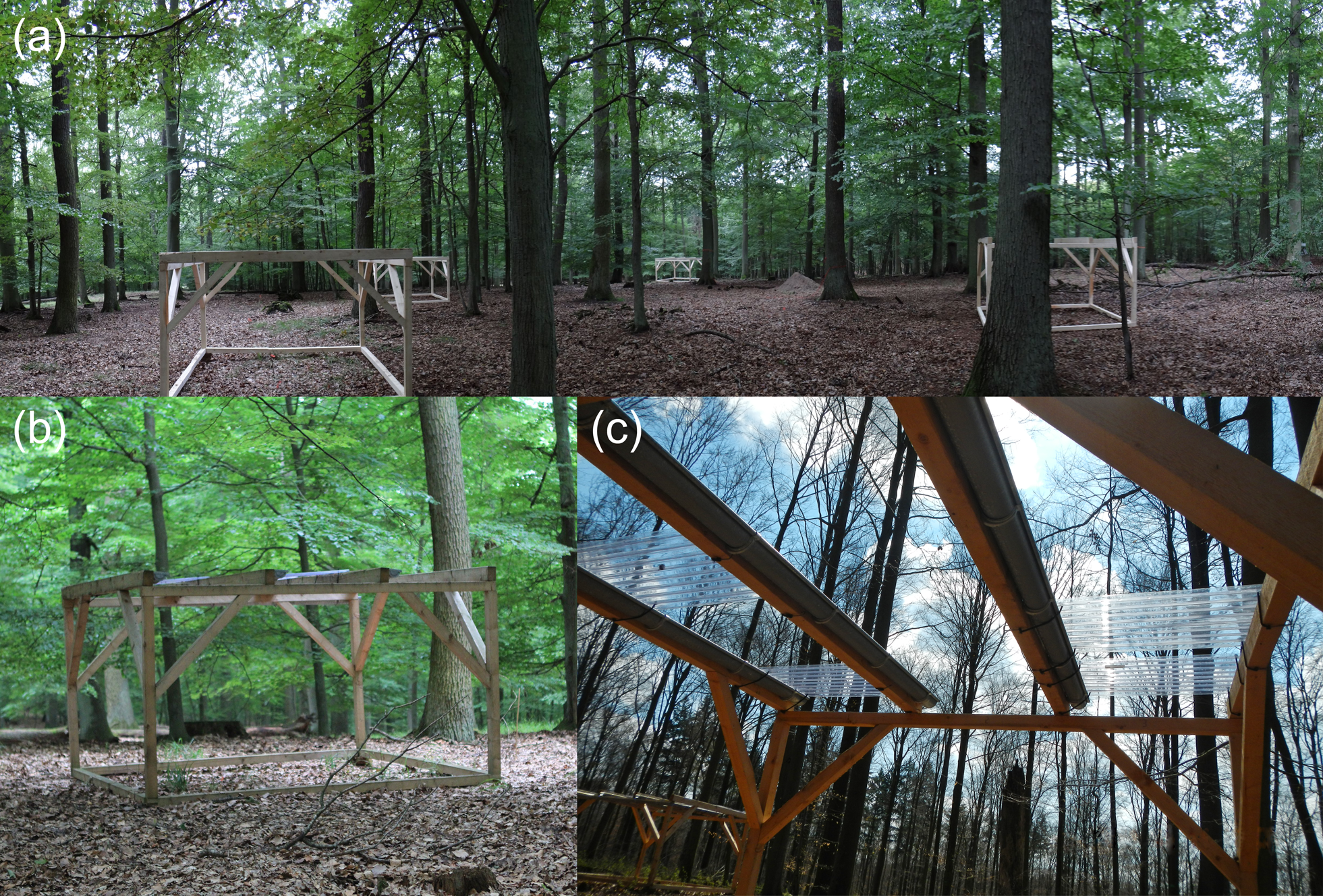

Supplement: S1 Fig — Assembly of the roofs (a), construction of a single roof (b) and a view indicating the acrylic transparent tiles and the rain gutters (c) are shown. Pictures were taken at the unmanaged beech plot of the Schorfheide-Chorin exploratory. (TIF) [file pone.0122539.s001.tif]
