# Supplementary material for: Soil Bacterial Community Structure Responses to Precipitation Reduction and Forest Management in Forest Ecosystems across Germany
Source: PLoS One. 2015 Apr 14;10(4):e0122539. doi: 10.1371/journal.pone.0122539 (PMC4397059; doi:10.1371/journal.pone.0122539)
Supplement: S1 Table — (DOCX) [file pone.0122539.s002.docx]

**Table S1. List of samples and barcodes.**

| Site | Plot ID | Sample | Barcode^a^ | HQ reads^b^ |
| --- | --- | --- | --- | --- |
| Schorfheide | ScmC | pS16C1 | AGGCGG | 9511 |
|  | ScmC | pS16C2 | CGGTAT | 8437 |
|  | ScmC | pS16C3 | TGACGA | 7762 |
|  | ScmC | pS16C4 | ACAAGG | 10232 |
|  | ScmR | pS16D1 | CCGTTCA | 9892 |
|  | ScmR | pS16D2 | CAAGAA | 8741 |
|  | ScmR | pS16D3 | AGTTGG | 9212 |
|  | ScmR | pS16D4 | TATCAA | 10917 |
|  | SbuC | pS48C1 | AACCAG | 8751 |
|  | SbuC | pS48C2 | TTCGAG | 8463 |
|  | SbuC | pS48C3 | AAGGTG | 7541 |
|  | SbuC | pS48C4 | TCTTGG | 7951 |
|  | SbuC | pS48D1 | AGACCT | 9058 |
|  | SbuR | pS48D2 | ATACCA | 8305 |
|  | SbuR | pS48D3 | TCGCGG | 3933 |
|  | SbuR | pS48D4 | ATCTTA | 11470 |
|  | SbmC | pS49C1 | AGTCGA | 8838 |
|  | SbmC | pS49C2 | ACGGCT | 8006 |
|  | SbmC | pS49C3 | TGCGTT | 12421 |
|  | SbmC | pS49C4 | TCTCGA | 11803 |
|  | SbmR | pS49D1 | TAATCT | 7669 |
|  | SbmR | pS49D2 | TCACCT | 11418 |
|  | SbmR | pS49D3 | TCCGCT | 11460 |
|  | SbmR | pS49D4 | TATTGA | 8546 |
| Schwäbische Alb | AbuC | pA08C1 | ACTAATT | 7904 |
|  | AbuC | pA08C2 | TGACCGT | 8101 |
|  | AbuC | pA08C3 | TGTCGGA | 4828 |
|  | AbuR | pA08D1 | TTGACAA | 12503 |
|  | AbuR | pA08D2 | TCCAGAA | 7124 |
|  | AbuR | pA08D4 | AAGGCCT | 4625 |
|  | AcmC | pA13C1 | TTGGAGG | 11453 |
|  | AcmC | pA13C2 | TTATCGG | 8748 |
|  | AcmC | pA13C3 | AAGAAGA | 8018 |
|  | AcmC | pA13C4 | AACTGTT | 8970 |
|  | AcmR | pA13D1 | ACGAGAA | 8942 |
|  | AcmR | pA13D2 | TGGTGAA | 7636 |
|  | AcmR | pA13D3 | TCGTTGT | 9641 |
|  | AcmR | pA13D4 | TTGTGTT | 10079 |
|  | AbmC | pA29C1 | AGTCCGT | 8452 |
|  | AbmC | pA29C2 | TTGAACT | 9705 |
|  | AbmC | pA29C3 | CGGTCTT | 9807 |
|  | AbmC | pA29C4 | AGGTTGT | 8911 |
|  | AbmR | pA29D1 | TTCTCAA | 9474 |
|  | AbmR | pA29D2 | CTTCCTT | 9125 |
|  | AbmR | pA29D3 | ATTCGTA | 7811 |
|  | AbmR | pA29D4 | CCTTCCG | 7577 |
|  | HcmC | pH03C1 | CGTCGT | 6770 |
| Hainich | HcmC | pH03C2 | AAGGCA | 5256 |
|  | HcmC | pH03C3 | AACAACT | 7067 |
|  | HcmC | pH03C4 | ACACGGA | 5812 |
|  | HcmR | pH03D1 | CCAGGA | 4532 |
|  | HcmR | pH03D2 | ACTCCT | 3991 |
|  | HcmR | pH03D3 | TTCCTG | 6144 |
|  | HcmR | pH03D4 | TTCATA | 6499 |
|  | HbuC | pH12C1 | AATGGTA | 11934 |
|  | HbuC | pH12C3 | AACCTGG | 9808 |
|  | HbuC | pH12C4 | ACGAAGT | 17333 |
|  | HbuR | pH12D1 | TGCCGAA | 10380 |
|  | HbuR | pH12D2 | TATTCGT | 18454 |
|  | HbuR | pH12D3 | TAGGAAT | 14059 |
|  | HbuR | pH12D4 | CCGGCCA | 10911 |
|  | HbmC | pH47C1 | TTCGCGA | 10637 |
|  | HbmC | pH47C2 | AACGAGG | 7318 |
|  | HbmC | pH47C3 | ACCTGAA | 10187 |
|  | HbmC | pH47C4 | AAGAGTT | 10797 |
|  | HbmR | pH47D1 | TTCGTGG | 8917 |
|  | HbmR | pH47D2 | AACACAA | 9893 |
|  | HbmR | pH47D3 | TTCTTGA | 10430 |
|  | HbmR | pH47D4 | TCCAAGT | 11110 |

^a^  Forward primer sequence: “A” adapter oligo sequence (CCATCTCATCCCTGCGTGTCT­CCG­ACTCAG) + barcode + linker (CG) + 534R (ATTACCGCGGCTGCTGG))

Reverse primer sequence: “B” adapter oligo sequence (CCTATCCCCTGTGTGCCTTGGCAG­TCTCAG) +8F (AGAGTTTGATCMTGGCTCAG)

^b^ Number of high-quality reads
