# Supplementary material for: Soil Bacterial Community Structure Responses to Precipitation Reduction and Forest Management in Forest Ecosystems across Germany
Source: PLoS One. 2015 Apr 14;10(4):e0122539. doi: 10.1371/journal.pone.0122539 (PMC4397059; doi:10.1371/journal.pone.0122539)
Supplement: S2 Table — (DOCX) [file pone.0122539.s003.docx]

**Table S2. Physical-chemical soil properties for each of the subplots.**

| Plot ID | Management type | Treatment | pH | Corg (%) | Nt (%) |
| --- | --- | --- | --- | --- | --- |
| Sbu | unmanaged | Control | 3.61 ± 0.06 | 4.04 ± 0.08 | 0.23 ± 0.10 |
|  |  | Roof | 3.90 ± 0.16 | 2.56 ± 0.10 | 0.15 ± 0.00 |
| Sbm | managed | Control | 3.52 ± 0.02 | 6.34 ± 0.23 | 0.34 ± 0.01 |
|  |  | Roof | 3.65 ± 0.09 | 6.98 ± 0.62 | 0.36 ± 0.03 |
| Scm | intensively managed | Control | 3.24 ± 0.01 | 6.23 ± 0.37 | 0.31 ± 0.02 |
|  |  | Roof | 3.31 ± 0.16 | 9.30 ± 0.85 | 0.47 ± 0.03 |
| Hbu | unmanaged | Control | 4.86 ± 0.09 | 4.07 ± 0.06 | 0.30 ± 0.00 |
|  |  | Roof | 4.33 ± 0.05 | 3.02 ± 0.07 | 0.23 ± 0.01 |
| Hbm | managed | Control | 5.18 ± 0.17 | 4.52 ± 0.11 | 0.33 ± 0.01 |
|  |  | Roof | 4.64 ± 0.12 | 4.43 ± 0.30 | 0.33 ± 0.02 |
| Hcm | intensively managed | Control | 3.81 ± 0.08 | 5.27 ± 0.26 | 0.34 ± 0.01 |
|  |  | Roof | 3.85 ± 0.05 | 6.03 ± 0.34 | 0.39 ± 0.02 |
| Abu | unmanaged | Control | 4.97 ± 0.10 | 8.76 ± 0.16 | 0.59 ± 0.01 |
|  |  | Roof | 4.99 ± 0.00 | 8.01 ± 0.85 | 0.55 ± 0.05 |
| Abm | managed | Control | 4.87 ± 0.13 | 7.12 ± 0.29 | 0.62 ± 0.00 |
|  |  | Roof | 4.96 ± 0.10 | 7.11 ± 0.26 | 0.56 ± 0.00 |
| Acm | intensively managed | Control | 3.85 ± 0.05 | 9.25 ± 0.11 | 0.47 ± 0.01 |
|  |  | Roof | 4.65 ± 0.11 | 8.31 ± 0.12 | 0.46 ± 0.02 |

pH, total nitrogen (N_t_) and organic carbon content (C_org_) of reduced precipitation and control subplots are provided as mean ± standard error of means.
