# Supplementary material for: Soil Bacterial Community Structure Responses to Precipitation Reduction and Forest Management in Forest Ecosystems across Germany
Source: PLoS One. 2015 Apr 14;10(4):e0122539. doi: 10.1371/journal.pone.0122539 (PMC4397059; doi:10.1371/journal.pone.0122539)
Supplement: S3 Table — (DOCX) [file pone.0122539.s004.docx]

**Table S3. Mean number of phylotypes at the five different taxonomic levels obtained for each of the subplots.**

| Plot ID | Management type | Treatment | Mean number of phylotypes | | | | |
| --- | --- | --- | --- | --- | --- | --- | --- |
|  |  |  | phyla | classes | orders | families | genera |
| Sbu | unmanaged | Control | 13 | 35 | 51 | 85 | 119 |
|  |  | Roof | 13 | 36 | 53 | 93 | 137 |
| Sbm | managed | Control | 14 | 44 | 60 | 108 | 158 |
|  |  | Roof | 14 | 41 | 57 | 104 | 155 |
| Scm | intensively managed | Control | 13 | 35 | 49 | 78 | 106 |
|  |  | Roof | 11 | 31 | 44 | 75 | 109 |
| Hbu | unmanaged | Control | 13 | 44 | 63 | 109 | 175 |
|  |  | Roof | 14 | 45 | 65 | 113 | 177 |
| Hbm | managed | Control | 14 | 45 | 64 | 111 | 176 |
|  |  | Roof | 14 | 45 | 63 | 111 | 171 |
| Hcm | intensively managed | Control | 11 | 34 | 48 | 85 | 113 |
|  |  | Roof | 12 | 38 | 54 | 95 | 137 |
| Abu | unmanaged | Control | 14 | 47 | 66 | 115 | 166 |
|  |  | Roof | 15 | 48 | 67 | 117 | 175 |
| Abm | managed | Control | 13 | 45 | 62 | 112 | 174 |
|  |  | Roof | 14 | 47 | 65 | 115 | 179 |
| Acm | intensively managed | Control | 13 | 40 | 58 | 100 | 141 |
|  |  | Roof | 13 | 43 | 63 | 109 | 162 |
