# Supplementary material for: Soil Bacterial Community Structure Responses to Precipitation Reduction and Forest Management in Forest Ecosystems across Germany
Source: PLoS One. 2015 Apr 14;10(4):e0122539. doi: 10.1371/journal.pone.0122539 (PMC4397059; doi:10.1371/journal.pone.0122539)
Supplement: S4 Table — (DOCX) [file pone.0122539.s005.docx]

**Table S4. Analysis of variance of the linear mixed effects models for the soil organic carbon content (C_org_), total nitrogen (N_t_) and pH.**

|  |  | **C_org_** | | | | | | **N_t_** | | | | | | **pH** | | | | | | | | |
| --- | --- | --- | --- | --- | --- | --- | --- | --- | --- | --- | --- | --- | --- | --- | --- | --- | --- | --- | --- | --- | --- | --- |
| Fixed effects^a^ | Num df | denDF^c^ | | F-value or z - score | | p^b^ | | denDF | | F-value or z - score | | p | | denDF | | | F-value or z - score | | | p | | |
| exploratory | 2 | 3.99 | | 4.295 | | 0.101 | | 3.99 | | 8.523 | | **0.036** | | 4 | | | 28.05 | | | **0.004** | | |
| Hainich - Alb |  |  | |  | |  | |  | | -3.536 | | **0.001** | |  | | | -1.57 | | | 0.351 | | |
| Schorfheide – Alb |  |  | |  | |  | |  | | -3.615 | | **0.001** | |  | | | -7.13 | | | **<.001** | | |
| Schorfheide - Hainich |  |  | |  | |  | |  | | -0.079 | | 1.000 | |  | | | -5.56 | | | **<.001** | | |
| management | 2 | 3.99 | | 1.284 | | 0.371 | | 3.99 | | 0.997 | | 0.445 | | 4 | | | 3.505 | | | **0.023** | | |
| managed - intensive |  |  | |  | |  | |  | |  | |  | |  | | |  | | |  | | |
| unmanaged - intensive |  |  | |  | |  | |  | |  | |  | |  | | |  | | |  | | |
| unmanaged - managed |  |  | |  | |  | |  | |  | |  | |  | | |  | | |  | | |
| treatment | 1 | 33.0 | | 0.001 | | 0.971 | | 33.1 | | 0.192 | | 0.664 | | 60 | | | 0.457 | | | 0.502 | | |
| precipitation reduction - control |  |  | |  | |  | |  | |  | |  | |  | | |  | | |  | | |
| management : treatment | 2 | 33.0 | | 11.26 | | **<.001** | | 33.1 | | 12.057 | | **<.001** | | 60 | | | 11.3 | | | **0.036** | | |
| cm_r - cm_c |  |  | | -0.016 | | 1.000 | |  | | -0.578 | | 1.000 | |  | | | 5.128 | | | **<.001** | | |
| bm_c - cm_c |  |  | | 2.013 | | 0.662 | |  | | 2.986 | | **0.042** | |  | | | 3.093 | | | **0.013** | | |
| bm_r - cm_c |  |  | | 1.125 | | 1.000 | |  | | 1.670 | | 1.000 | |  | | | 3.335 | | | **0.011** | | |
| bu_c - cm_c |  |  | | 1.565 | | 1.000 | |  | | 2.277 | | 0.342 | |  | | | 3.380 | | | **0.008** | | |
| bu_r - cm_c |  |  | | 0.786 | | 1.000 | |  | | 1.533 | | 1.000 | |  | | | 3.448 | | | 1.000 | | |
| bm_c - cm_r |  |  | | 2.020 | | 0.651 | |  | | 3.239 | | **0.018** | |  | | | 0.694 | | | 1.000 | | |
| bm_r - cm_r |  |  | | 1.132 | | 1.000 | |  | | 1.923 | | 0.817 | |  | | | 0.936 | | | 1.000 | | |
| bu_c - cm_r |  |  | | 1.572 | | 1.000 | |  | | 2.530 | | 0.171 | |  | | | 0.981 | | | 1.000 | | |
| bu_r - cm_r |  |  | | 0.793 | | 1.000 | |  | | 1.781 | | 1.000 | |  | | | 1.049 | | | 1.000 | | |
| bm_r - bm_c |  |  | | -2.016 | | 0.658 | |  | | -3.004 | | **0.040** | |  | | | 0.516 | | | 1.000 | | |
| bu_c - bm_c |  |  | | -0.448 | | 1.000 | |  | | -0.708 | | 1.000 | |  | | | 0.287 | | | 1.000 | | |
| bu_r - bm_c |  |  | | -1.184 | | 1.000 | |  | | -1.389 | | 1.000 | |  | | | 0.355 | | | 1.000 | | |
| bu_c - bm_r |  |  | 0.440 | | 1.000 | |  | | 0.607 | | 1.000 | |  | | 0.045 | | | 1.000 | | |  |  |
| bu_r - bm_r |  |  | -0.315 | | 1.000 | |  | | -0.101 | | 1.000 | |  | | 0.113 | | | 1.000 | | |  |  |
| bu_r - bu_c |  |  | -1.562 | | 1.000 | |  | | -1.463 | | 1.000 | |  | | | 0.145 | | | 1.000 | | |  |

^a^ Linear mixed effects models were conducted for soil parameters as a function of exploratory, management intensity and treatment (precipitation reduction). Results of multiple comparisons of means (Tukey contrasts) were calculated for each parameter per factor of the linear mixed effects models. P values were adjusted by the Bonferroni method. Thus, since tested parameters were affected by an interaction of management and treatment, post-hoc tests were applied for treatment within management and not for management and treatment alone.

^b^ Significant probabilities (< 0.05) are shown in bold.

^c^ denDF is the number of degrees of freedom associated with the model errors.
cm = intensive conifer managed, bm = beech managed, bu = beech unmanaged,
r = precipitation reduction subplot, c = control subplot
