# Supplementary material for: Soil Bacterial Community Structure Responses to Precipitation Reduction and Forest Management in Forest Ecosystems across Germany
Source: PLoS One. 2015 Apr 14;10(4):e0122539. doi: 10.1371/journal.pone.0122539 (PMC4397059; doi:10.1371/journal.pone.0122539)
Supplement: S5 Table — (DOCX) [file pone.0122539.s006.docx]

**Table S5. Analysis of variance of the linear mixed effects models for the understorey diversity parameters.**

|  |  | **LAI_sp_** | | | | **species richness** | | | **H’** | | |
| --- | --- | --- | --- | --- | --- | --- | --- | --- | --- | --- | --- |
| Fixed factors^a^ | Num df | | denDF^c^ | F-value or z - score | p^b^ | denDF | F-value or z - score | p | denDF | F-value or z - score | p |
| exploratory | 2 | | 3 | 2.4125 | 0.237 | 3 | 15.445 | **0.026** | 56 | 17.35 | **<.001** |
| Hainich - Alb |  | |  | -2.001 | 0.136 |  | -3.308 | **0.003** |  | -3.9 | **<.001** |
| Schorfheide – Alb |  | |  | -1.674 | 0.283 |  | -5.450 | **<.001** |  | -5.62 | **<.001** |
| Schorfheide - Hainich |  | |  | 0.033 | 1.000 |  | -2.629 | **0.026** |  | -2.21 | 0.080 |
| management | 2 | | 3 | 1.8393 | 0.301 | 3 | 18.113 | **0.021** | 56 | 16.12 | **<.001** |
| managed - intensive |  | |  | -1.535 | 0.375 |  | 1.305 | 0.5756 |  | 1.551 | 0.363 |
| unmanaged - intensive |  | |  | -1.823 | 0.205 |  | -2.950 | **0.010** |  | -1.85 | 0.194 |
| unmanaged - managed |  | |  | -0.511 | 1.000 |  | -4.080 | **<.001** |  | -3.20 | **0.004** |
| treatment | 1 | | 29 | 6.3146 | **0.018** | 53 | 0.2813 | 0.598 | 56 | 0.093 | 0.761 |
| precipitation reduction - control |  | |  | -2.387 | **0.017** |  | 0.198 | 0.843 |  | 0.172 | 0.863 |
| management : treatment | 2 | | 29 | 2.0267 | 0.150 | 53 | 1.2305 | 0.300 | 56 | 0.899 | 0.413 |

^a^ Linear mixed effects models were conducted for soil parameters as a function of exploratory, management intensity and treatment (precipitation reduction). Results of multiple comparisons of means (Tukey contrasts) were calculated for each parameter per factor of the linear mixed effects models. P values were adjusted by the Bonferroni method.

^b^ Significant probabilities (< 0.05) are shown in bold.

^c^ LAIsp = leaf area index, H’ = Shannon’s diversity index,
denDF = number of degrees of freedom associated with the model errors,
cm = intensive conifer managed, bm = beech managed, bu = beech unmanaged,
r = precipitation reduction subplot, c = control subplot
