# Supplementary material for: Soil Bacterial Community Structure Responses to Precipitation Reduction and Forest Management in Forest Ecosystems across Germany
Source: PLoS One. 2015 Apr 14;10(4):e0122539. doi: 10.1371/journal.pone.0122539 (PMC4397059; doi:10.1371/journal.pone.0122539)
Supplement: S6 Table — (DOCX) [file pone.0122539.s007.docx]

**Table S6. Analysis of variance of the linear mixed effects models for the diversity parameters of the pyrosequencing data.**

^

|  |  | | **OTU richness** | | | **Inverse Simpson** | | | | | **H** | | | **No. of phylotypes** | | | | | | |  |
| --- | --- | --- | --- | --- | --- | --- | --- | --- | --- | --- | --- | --- | --- | --- | --- | --- | --- | --- | --- | --- | --- |
| Fixed factors^a^ | Num df | denDF^c^ | | F-value or z - score | p^b^ | | denDF | F-value or z - score | p | denDF | | F-value or z - score | p | denDF | | F-value or z - score | | p | |  |  |
| exploratory | 2 | 4.25 | | 21.166 | **0.006** | | 5.02 | 8.1137 | **0.027** | 3.93 | | 16.110 | **0.013** | 4.0 | | 8.119 | | **0.039** | |  |  |
| Hainich - Alb |  |  | | -3.400 | **0.002** | |  | -3.311 | **0.003** |  | | -3.652 | **<.001** |  | | -0.91 | | **1.000** | |  |  |
| Schorfheide – Alb |  |  | | -6.505 | **<.001** | |  | -3.039 | **0.007** |  | | -5.596 | **<.001** |  | | -3.85 | | **<.001** | |  |  |
| Schorfheide - Hainich |  |  | | -3.107 | **0.006** | |  | 0.276 | 1.000 |  | | -1.994 | 0.156 |  | | -2.94 | | **0.01** | |  |  |
| management | 2 | 28.0 | | 29.79 | **<.001** | | 22 | 3.2379 | 0.086 | 3.93 | | 16.890 | **0.012** | 4.0 | | 10.49 | | **0.026** | |  |  |
| managed - intensive |  |  | |  |  | |  | 1.315 | 0.566 |  | | 2.998 | **0.008** |  | |  | |  | |  |  |
| unmanaged - intensive |  |  | |  |  | |  | 1.955 | 0.152 |  | | 5.881 | **<.001** |  | |  | |  | |  |  |
| unmanaged - managed |  |  | |  |  | |  | 0.652 | 1.000 |  | | 2.942 | **0.010** |  | |  | |  | |  |  |
| treatment | 1 | 33.2 | | 1.1825 | 0.285 | | 33.1 | 0.0117 | 0.915 | 31.3 | | 3.621 | 0.066 | 32 | | 13.35 | | **0.001** | |  |  |
| precipitation reduction - control |  |  | |  |  | |  |  |  |  | |  |  |  | |  | |  | |  |  |
| management * treatment | 2 | 32.9 | | 4.668 | **0.038** | | 32.7 | 0.5954 | 0.446 | 31.3 | | 1.283 | 0.291 | 32 | | 5.016 | | **0.013** | |  |  |
| cm_r - cm_c |  |  | | 4.361 | **<.001** | |  |  |  |  | |  |  |  | | 3.429 | | **0.009** | |  |  |
| bm_c - cm_c |  |  | | 3.492 | **0.008** | |  |  |  |  | |  |  |  | | 2.399 | | 0.247 | |  |  |
| bm_r - cm_c |  |  | | 3.826 | **0.002** | |  |  |  |  | |  |  |  | | 2.726 | | 0.096 | |  |  |
| bu_c - cm_c |  |  | | 3.756 | **0.003** | |  |  |  |  | |  |  |  | | 1.805 | | 1.000 | |  |  |
| bu_r - cm_c |  |  | | 4.046 | **0.001** | |  |  |  |  | |  |  |  | | 2.422 | | 0.232 | |  |  |
| bm_c - cm_r |  |  | | 1.598 | 1.000 | |  |  |  |  | |  |  |  | | 0.854 | | 1.000 | |  |  |
| bm_r - cm_r |  |  | | 1.932 | 0.800 | |  |  |  |  | |  |  |  | | 1.181 | | 1.000 | |  |  |
| bu_c - cm_r |  |  | | 1.908 | 0.846 | |  |  |  |  | |  |  |  | | 0.291 | | 1.000 | |  |  |
| bu_r - cm_r |  |  | | 2.198 | 0.420 | |  |  |  |  | |  |  |  | | 0.908 | | 1.000 | |  |  |
| bm_r - bm_c |  |  | | 0.770 | 1.000 | |  |  |  |  | |  |  |  | | 0.726 | | 1.000 | |  |  |
| bu_c - bm_c |  |  | | 0.349 | 1.000 | |  |  |  |  | |  |  |  | | -0.55 | | 1.000 | |  |  |
| bu_r - bm_c |  |  | | 0.639 | 1.000 | |  |  |  |  | |  |  |  | | 0.071 | | 1.000 | |  |  |
| bu_c - bm_r |  |  | | 0.022 | 1.000 | |  |  |  |  | |  |  | |  | | -0.87 | | 1.000 | | |
| bu_r - bm_r |  |  | | 0.312 | 1.000 | |  |  |  |  | |  |  | |  | | -0.25 | | 1.000 | | |
| bu_r - bu_c |  |  | | 0.592 | 1.000 | |  |  |  |  | |  |  | |  | | 1.211 | | 1.000 | | |

^a^ Linear mixed effects models were conducted for diversity parameters as a function of exploratory, management intensity and treatment (precipitation reduction). Results of multiple comparisons of means (Tukey contrasts) were calculated for each parameter per factor of the linear mixed effects models. P values were adjusted by the bonferroni method. Thus, since tested parameters were affected by an interaction of management and treatment, post-hoc tests were applied for treatment within management and not for management and treatment alone.

^b^ Significant probabilities (< 0.05) are shown in bold.

^

^c^ H = non-parametric estimate of the classical Shannon’s diversity index; denDF = number of degrees of freedom associated with the model errors; cm = intensive conifer managed, bm = beech managed, bu = beech unmanaged, r = precipitation reduction subplot, c = control subplot
